# Role of *P27 *-*P55 *operon from *Mycobacterium tuberculosis *in the resistance to toxic compounds

**DOI:** 10.1186/1471-2334-11-195

**Published:** 2011-07-16

**Authors:** María V Bianco, Federico C Blanco, Belén Imperiale, Marina A Forrellad, Roxana V Rocha, Laura I Klepp, Angel A Cataldi, Nora Morcillo, Fabiana Bigi

**Affiliations:** 1 Instituto de Biotecnología, CICVyA-INTA, N. Repetto and De los Reseros, 1686 Hurlingham, Buenos Aires, Argentina; 2 Reference Laboratory of Tuberculosis Control Program of Buenos Aires Province, Dr Cetrángolo Hospital, Buenos Aires, Argentina

**Keywords:** *Mycobacterium tuberculosis*, *lprG*, *P55*, *P27*

## Abstract

**Background:**

The *P27-P55 *(*lprG-Rv1410c*) operon is crucial for the survival of *Mycobacterium tuberculosis*, the causative agent of human tuberculosis, during infection in mice. *P55 *encodes an efflux pump that has been shown to provide *Mycobacterium smegmatis *and *Mycobacterium bovis *BCG with resistance to several drugs, while *P27 *encodes a mannosylated glycoprotein previously described as an antigen that modulates the immune response against mycobacteria. The objective of this study was to determine the individual contribution of the proteins encoded in the *P27-P55 *operon to the resistance to toxic compounds and to the cell wall integrity of *M. tuberculosis*.

**Method:**

In order to test the susceptibility of a mutant of *M. tuberculosis *H37Rv in the *P27-P55 *operon to malachite green, sodium dodecyl sulfate, ethidium bromide, and first-line antituberculosis drugs, this strain together with the wild type strain and a set of complemented strains were cultivated in the presence and in the absence of these drugs. In addition, the malachite green decolorization rate of each strain was obtained from decolorization curves of malachite green in PBS containing bacterial suspensions.

**Results:**

The mutant strain decolorized malachite green faster than the wild type strain and was hypersensitive to both malachite green and ethidium bromide, and more susceptible to the first-line antituberculosis drugs: isoniazid and ethambutol. The pump inhibitor reserpine reversed *M. tuberculosis *resistance to ethidium bromide. These results suggest that P27-P55 functions through an efflux-pump like mechanism. In addition, deletion of the *P27-P55 *operon made *M. tuberculosis *susceptible to sodium dodecyl sulfate, suggesting that the lack of both proteins causes alterations in the cell wall permeability of the bacterium. Importantly, both P27 and P55 are required to restore the wild type phenotypes in the mutant.

**Conclusions:**

The results clearly indicate that P27 and P55 are functionally connected in processes that involve the preservation of the cell wall and the transport of toxic compounds away from the cells.

## Background

Infection by *Mycobacterium tuberculosis *is a major health problem worldwide [1]. Pathogenic mycobacterial species show remarkable ability to survive in the diverse conditions encountered during the infection process [2]. However, even after decades of investigation, the knowledge about the mycobacterial pathogenesis remains insufficient. The identification of the genes associated with the multiplication and survival of bacilli in the host has provided a framework to study *M. tuberculosis *virulence [3]. However, little is still known about the role of the encoded products in the interaction between the host and the pathogen. Elucidating these functions is then the next main challenge in tuberculosis research.

We have previously demonstrated that *P27-P55 *(*lprG-Rv1410c*) operon [4] is crucial for the survival of *M. tuberculosis *during infection in mice [5]. *P55 *encodes for the efflux pump, which has been shown to provide resistance to several drugs, likely through a process coupled to oxidative balance within the cell [6]. Also, it has been demonstrated that over-expression of P55 from *M. tuberculosis *in *M. smegmatis *confers resistance to several compounds by an efflux pumping activity [7]. *P27*, on the other hand, encodes a lipoprotein previously described as an antigen in the *M. tuberculosis *complex [8] and, as many other mycobacterial lipoproteins, P27 is a mannosylated glycoprotein [9].

Although several publications have characterized the proteins encoded in the *P27-P55 *operon [4-14], the mechanism by which this operon contributes to the virulence of *M. tuberculosis *is still unclear. The finding that P27 causes an adverse effect when used as a protein-based vaccine [15], together with the fact that this glycolipoprotein inhibits MHC-II Ag processing, suggests that P27 acts modulating the immune response against mycobacteria (as an evasion mechanism) in favour of bacterial persistence [16].

Increasing evidence indicates that mycobacterial lipoproteins are involved in cell wall integrity either maintaining cell wall permeability [17] or participating in cell wall synthesis with specific functions [18]. Recently, it has been shown that P27 acts in cooperation with P55 to transport ethidium bromide in *M. smegmatis *[10], indicating that P27 is necessary for P55-mediated transport across the cell membrane. Moreover, it has been recently proposed that in mycobacteria, P27 functions as a carrier of glycolipids during their trafficking and delivery to the mycobacterial cell wall [11].

In order to gain more insight into the function of the *P27-P55 *operon, in the present work, we studied the contribution of both P27 and P55 on the resistance to toxic compounds as well as on the cell wall integrity of *M. tuberculosis*.

## Methods

### Bacterial strains and culture media

All cloning steps were performed in *Escherichia coli *DH5α. *E. coli *was grown either in Luria-Bertani (LB) broth or on LB agar. *Mycobacterium *strains were grown in Middlebrook 7H9 medium supplemented with 0.05% Tween 80, albumin 0.5%, dextrose 0.4%, and 0.5% glycerol, or Middlebrook 7H10 supplemented with albumin 0.5%, dextrose 0.4%, and 0.5% glycerol. When necessary, 50 μg/ml hygromycin, 20 μg/ml kanamycin or 20 μg/ml reserpine were added to the media. Electrocompetent cells of the previously obtained *M. tuberculosis *ΔP27 (MtΔP27) mutant [5] were prepared following the procedure described in [5]. For culture supernatant protein preparations, strains were cultured in Sauton supplemented with 0.5% glycerol.

### Construction of ΔP27 *M. tuberculosis *complemented strains

Complemented strains of the MtΔP27 mutant [5] expressing either P27 or P55 were generated in this study. The *P27 *gene under the control of the *P27-P55 *operon promoter was cloned into the pNBV1 vector [19]. pNBV1 was also used as a backbone vector to clone the *P55 *gene under the *hsp60 *promoter. These plasmids together with plasmid ΔP27C, which express the *P27-P55 *operon under its own promoter in the pNBV1 backbone [5], were used to transform MtΔP27 by electroporation [5]. The resulting complemented strains are referred to as MtΔP27::P27, MtΔP27::P55 and MtΔP27::P27-P55, respectively.

### Ethidium bromide sensitivity assays

*M. tuberculosis *strains were grown in liquid medium in the presence of either 1 μg/ml or 0.5 μg/ml of ethidium bromide. When necessary, 20 μg/ml reserpine was added into de cultures. Bacterial growth was monitored by optical density (OD) and compared to growth in the absence of ethidium bromide.

### Malachite green decolorization assay

The malachite decolorization assay was performed as described in Banaei *et al*. [17]. Briefly, malachite green (final concentration of 0.1 mg/liter) was added to 4 ml of mid-log bacterial cultures resuspended in phosphate-buffered saline (PBS) to an OD of 0.5 at 600 nm. When necessary, 20 μg/ml reserpine was added into de cultures. Bacterial suspensions were immediately centrifuged and the absorbance at 620 nm was measured in the supernatants at 10-min intervals. Because of the photooxidation of malachite green, tubes were covered with foil and the experiments were performed under reduced light. The experiment was repeated three times. The decolorization rates were expressed as nanograms of dye decolorized every 10 min per ml of culture.

### Malachite green and SDS sensitivity assays

Two methods were used to determine the susceptibilities of the *M. tuberculosis *strains to sodium dodecyl sulfate (SDS). Firstly, a disc assay was used to determine the inhibition of growth in the presence of SDS. Briefly, bacterial suspensions containing 10^7 ^cells were spread on Middlebrook 7H10 agar plates, and discs containing 10 μl of 10% SDS were placed in the middle of the plate. Halos were recorded after two weeks at 37°C.

Secondly, the method described by Banaei *et al*. [17] was used to determine the loss of bacterial viability in the presence of a high concentration of SDS. Briefly, bacterial cultures grown to mid-log phase were diluted with growth medium to an OD of 0.05 at a wavelength of 600 nm and incubated with SDS 0.05% in duplicate. At 1 and 4 h, bacterial CFU were counted on Middlebrook 7H10 agar plates.

The malachite green sensitivity assay was performed as follows: bacterial suspensions with 10^6 ^cells were plated on Middlebrook 7H10 agar with or without 1 mg/liter of malachite green. Plates were incubated at 37°C in the dark and CFUs were counted after 21 days in plates without malachite green and after 40 days in plates containing malachite green.

### Protein preparations, SDS-PAGE and Western blots

Subcellular fractions of *Mycobacterium *strains were obtained as previously described [8]. Proteins were separated in SDS-PAGE and transferred to nitrocellulose filters. Western blot assays were performed as previously described [8] with the antibody anti-P27 Mab antibody (1:500). Alkaline phosphatase-conjugated anti-mouse immunoglobulin G (1:10000/Sigma-Aldrich) was used as secondary antibody.

### First-line drug susceptibility testing (DST) by the colorimetric microplate-based method

To determine the minimal inhibitory concentration (MIC) of anti-tuberculosis drugs, a non commercial, microplate colorimetric-based method with resazurin (REMA) was used as a general indicator of cellular growth and viability, following a previously described method [20-22]. Briefly, a 96 wells, microtiter, flat bottom plate was used to perform DST to isoniazid, rifampicin and ethambutol. When necessary, 20 μg/ml reserpine was added into de cultures. Serial twofold dilutions of the drugs were performed and wells were left free of drugs to be used as growth controls. Wells were inoculated with 100 μl of 1:25 from a bacterial suspension with turbidity comparable to 1.0 MacFarland standard (original bacterial suspension). Plates were incubated for 5 days at 37°C at normal atmosphere. After the incubation period, 0.02% of resazurin was added to the wells and incubated for 24 h. The MIC for each particular drug was considered as the lowest concentration showing less change of colour than the growth controls.

### Statistical analysis

The data were analyzed with Microsoft Excel statistical software using Student *t *test. *p *values <0.05 were considered statistically significant.

## Results

### P27 localizes in the cell wall, cell membrane and culture supernatant

**Figure 1:**
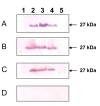
**Cellular localization of P27**. SDS-PAGE and western blot analysis of culture supernatant proteins (A), cell wall proteins (B), cell membrane proteins (C) and cytoplasm proteins (D) from MtΔP27 (lane 1), H37Rv (lane 2), MtΔP27::P27 (lane 3), MtΔP27::P27-P55 (lane 4) and MtΔP27::P55 (lane 5). Bands were detected by incubation with monoclonal anti-P27 serum (1:500) followed by alkaline phosphatase conjugated anti-rabbit immunoglobulin G (1:2000).

Previously, we have shown that P27 localizes in the cell membrane of *Mycobacterium bovis *[8]. However, in a recent study, P27 was purified from culture supernatant of *M. tuberculosis *by binding to concanavalin A [9], indicating that P27 is secreted outside the bacterium. In order to confirm these previous findings we carried out a cellular localization study of P27. For this purpose, the localization of P27 was assessed in H37Rv as well as in the mutant MtΔP27, and in a set of complemented strains. MtΔP27 is a strain knockout in the *P27-P55 *operon generated by the insertion of the kanamycin resistance cassette into the *P27 *gene of *M. tuberculosis *H37Rv [5]. Protein fractions were obtained from MtΔP27, the complemented strains carrying a wild type copy of either *P27 *or *P55*, a full complemented strain transformed with the complete operon and the wild type strain. Western blotting using an anti-LprG antibody showed a 27-kDa band present in the cell wall, cell membrane and culture supernatant but absent in the cytoplasm fraction of the wild type and complemented strains, MtΔP27::P27 and MtΔP27::P27-P55. Therefore, the expression of P27 in both MtΔP27::P27 and MtΔP27::P27-P55 strains confirms the functionality and integrity of the plasmids used to complement the mutant strain (Figure 1). No band was detected in any of the protein fractions of the MtΔP27 mutant and MtΔP27::P55 complemented strain (Figure 1). These results indicate that P27 is secreted from the bacterium to the medium but also demonstrate that this protein is a component of the cell wall of *M. tuberculosis*. Expression of *P55 *in the wild type and complemented strains was confirmed by RT-qPCR (see additional file 1).

### Sensitivity of ΔP27 mutant to malachite green

**Figure 2:**
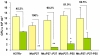
**Bacterial viability after exposure to malachite green**. Bacterial strains grown to mid-log phase were diluted to 10^7 ^cells/ml and 0.1 ml was plated on 7H10 medium with (green bars) or without (yellow bars) 1 mg/liter of malachite green. Data points show means +/- standard deviations of duplicate. Significantly different from values of H37Rv (*p *< 0.05)* (*p *< 0.01)**. All data are representative of two independent experiments.

It has been demonstrated that in the absence of the lipoprotein LspA, *M. tuberculosis *is more sensitive to malachite green, likely due to a cell wall permeability defect [17]. Thus, we decided to assess the contribution of both P27 and P55 to the resistance of *M. tuberculosis *to malachite green. For this purpose, equivalent densities of the wild type, the mutant and complemented strains were plated in media with and without malachite green. In the presence of malachite green, the MtΔP27 mutant showed 100% of reduction on CFU counts while the wild type and the full complemented strains exhibited 45.2% and 62.5% of inhibition, respectively, as compared to the growth in the absence of malachite green. Complementation of the MtΔP27 mutant with *P27 *or *P55 *alone reduced the level of inhibition to 90.2% and 81.9%, respectively (Figure 2). These results indicate that both P27 and P55 are essential for *M. tuberculosis *to resist to the toxic effect exerted by malachite green and that the only presence of P55 allows partial levels of resistance.

In order to gain insights into the mechanism involved in the resistance to malachite green we compared the ability of the MtΔP27 mutant to decolorize malachite green with those of the complemented and wild type strains. Table 1 (and additional file 2) shows that the MtΔP27 mutant decolorized malachite green at a rate greater than that of the wild-type strain. Importantly, the expression of either P27 or P55 in the mutant did not complement the wild type phenotype. The decolorization rate of the mutant strain complemented with both *P27 *and *P55 *was equivalent to that of the wild type strain, indicating that only the reintroduction of the complete operon restored the wild type phenotype of *M. tuberculosis*. To elucidate whether an efflux pump activity was the mechanism by which P27 and, more likely, P55 excluded malachite green from the bacteria, the decolorization rates were determined in cultures of *M. tuberculosis *and the complemented strains in the presence of reserpine, a multidrug resistance pump inhibitor. Unexpectedly, in the presence of subinhibitory concentration of reserpine, the decolorization rates of malachite green for all strains were equivalent and comparable to those of the wild type and full complemented strain, in the absence of reserpine. This result suggests that reserpine acts somehow inhibiting the mechanism that mediates the decolorization of malachite green by *M. tuberculosis*. In agreement with this presumption, it has been suggested that *Mycobacterium avium *decolorizes malachite green through a process that involves a membrane protein, likely cytochrome P-450, and that this process requires hydrogen ion transfer across the membrane [23]. However, whether or not reserpine can inhibit decolorization activity needs to be investigated.

**Table 1:** Rates of malachite green decolorization by *M.tuberculosis *ΔP27 strains

	Decolorization rate^§ ^+/- SD	
	Reserpine 20 μg/ml	
	-	+
Strains		
MtH37Rv	9.0 (0.01)	10.7 (0.74)
MtΔP27	15.3 (0.54)*	11.6 (0.34)
MtΔP27+P27	15.3 (0.54)*	12.3 (1.90)
MtΔP27+P55	13.1 (0.28)*	13.3 (1.82)
MtΔP27+P27+P55	9.5 (0.24)	11.9 (1.60)

**^§^**Rates are expressed as nanograms of malachite green decolorized in 10 min per milliliter of culture at 37°C. Values show means +/- standard deviations of duplicates. All data are representative of three independent experiments. *Significantly different from values of H37Rv (*p *< 0.005).

### The lack of P27 and P55 alters the cell wall permeability of *M. tuberculosis*

**Figure 3:**
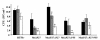
**Bacterial viability after exposure to SDS**. Bacterial strains grown to mid-log phase were diluted to an OD 600 nm of 0.05 in 7H9 medium containing 0.05% SDS and incubated at 37C for 0 h (black bars), 1 h (gray bars) and 4 h (white bars). Data points show means +/- standard deviations of duplicates. *Significantly different from values at T0 (*p *< 0.05). All data are representative of two independent experiments.

Drage and collaborators [11] have demonstrated that P27 binds glycolipids, which are central components of the mycobacterial cell wall. This suggests a role of the *P27-P55 *operon in the cell wall integrity of *M. tuberculosis*. To test this hypothesis, we assessed the impact of a mutation in the *P27-P55 *operon on the cell permeability of *M. tuberculosis*. To this end, we determined the susceptibility of the MtΔP27 to the detergent SDS. In the presence of SDS, the growth of the mutant strain was inhibited to a larger extent than that of the wild type (Table 2). The reintroduction of a wild type copy of the operon in the mutant strain partially restored the resistance of *M. tuberculosis *to SDS. Treatment of MtΔP27 with 0.05% SDS for 1 and 4 h resulted in 55% and 75% loss of viability, respectively (Figure 3). After 1 h of SDS treatment, the expression of either P55 or P27 partially restored (32% and 25% loss of viability) the wild type level of resistance to SDS in the mutant strain. The wild type and the double complemented strain were unaffected at 1 h and fairly affected at 4 h post treatment. Taken together these results demonstrate that the inactivation of the *P27-P55 *operon alters the sensitivity of *M. tuberculosis *to SDS, indicating that both proteins are required to maintain the integrity of the cell wall.

**Table 2:** Susceptibility of *M.tuberculosis *ΔP27 strains to SDS

Strain	Inhibition zone (cm) ^§^
H37Rv	2.43 (0.287)
MtΔP27	3.75 (0.354) *
MtΔP27+P27	3.44 (0.134) *
MtΔP27+P55	3.05 (0.071) *
MtΔP27+P27+P55	2.71 (0.103)

**^§^**Values show means +/- standard deviations of duplicates. All data are representative of three independent experiments. *Significantly different from values of H37Rv (*p *< 0.05).

### Sensitivity to isoniazid, ethambutol and ethidium bromide is increased in the ΔP27 mutant

To explore the possibility that the absence of P27 and P55 increases the susceptibility of *M. tuberculosis *to cell wall-targeting drugs, we tested the resistance of MtΔP27 to rifampicin, ethambutol and isoniazid. While the lack of P27 and P55 did not show to affect the resistance to rifampicin, mutant MtΔP27 was more susceptible to both isoniazid and ethambutol than the wild type strain. The wild type level of resistance to isoniazid and ethambutol was reversed when a copy of the whole operon was introduced in the mutant strain. However, the introduction of *P27 *or *P55 *alone did not complement the wild type level of resistance (Table 3), indicating that both genes are implicated in this mechanism. The addition of subinhibitory concentration of reserpine did not significantly affect the susceptibility of the wild type and full complemented strain to isoniazid and ethambutol (data not shown), which is consistent with previous reports [24,25].

**Table 3:** Antimicrobial susceptibilities of *M.tuberculosis *ΔP27 strains

MIC (mg/liter) for *M. tuberculosis *strain*					
	**MtH37Rv**	**MtΔP27**	**MtΔP27+P27**	**MtΔP27+P55**	**MtΔP27+P27+P55**

Rifampicin	0.06	0.06	0.06	0.06	0.06
Isoniazid	0.06	0.03	0.03	0.03	0.06
Ethambutol	2	1	1	1	2

*MICs were determined by the colorimetric microplate-based method.

**Figure 4:**
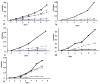
**Ethidium bromide susceptibility in the MtΔP27 mutant**. Bacterial strains grown to mid-log phase were diluted in 7H9 medium either with (blue and gray lines) or without (black lines) 1 μg/ml (full lines) or 0.5 μg/ml (dotted lines) of ethidium bromide and growth was determined over time as indicated. Assays were performed in the presence (blue lines) or absence (black and gray lines) of 20 μg/ml reserpine. Growth curves of **a**: H37Rv, **b**: MtΔP27 **c**: MtΔP27::P27, **d**: MtΔP27:: P55 and **e**: MtΔP27::P27-P55 are representative of two independent experiments. *Significantly different from values of H37Rv (*p *< 0.05).

Because it has been demonstrated that a mutant of *Mycobacterium smegmatis *in the homologous *P27-P55 *operon is more susceptible to ethidium bromide, and that drug resistance is restored by the intact operon from *M. tuberculosis *[10], we decided to investigate whether the *P27-P55 *operon provides *M. tuberculosis *resistance to ethidium bromide. MtΔP27 showed much higher sensitivity to both high and low concentrations of ethidium bromide than the wild type strain (Figure 4). Introduction of a wild type allele of the operon restored the ethidium bromide resistance to levels equivalent to those of the wild type strain. While complementation with *P27 *did not significantly modify the susceptibility of MtΔP27 to ethidium bromide, the introduction of *P55 *provided the mutant with partial resistance to low concentration of the drug. In the presence of reserpine, all strains were sensitive to ethidium bromide (Figure 4)

## Discussion

The increased sensitivity to membrane-perturbing compounds, such as SDS, observed in the mutant MtΔP27 is in agreement with a recent report demonstrating that P27 has a role in *M. tuberculosis *cell wall integrity by binding to glycolipids [11]. Other lipoproteins have also been demonstrated to have function related with cell wall of mycobacteria. Such is the example of LppX, a lipoprotein required for the translocation of phthiocerol dimycocerosates (DIM) to the outer membrane of *M. tuberculosis *[18]. Here we found evidences suggesting that P55 is also required to maintain the cell wall impermeability of *M. tuberculosis*, which is consistent with its cell membrane localization [7]. Although we have previously demonstrated that P27 localizes in the cell membrane fraction of *M. bovis *[8] here we showed that P27 is secreted to the culture supernatant of *M. tuberculosis*. However, a considerable amount of the protein was also detected in the mycobacterial cell wall, which is consistent with the proposed role of P27 in the transport of glycolipids such as lipomannans and lipoarabinomannans [11]. Therefore, the localization of P27 in both culture supernatant and cell wall fractions suggests that the alteration of the cell wall integrity detected in the mutant may be due not only to the mislocalization of P27 in the cell wall but also to the lack of some specific function exerted by the secreted P27. Regarding to this last aspect, the structure of P27 from *M. tuberculosis *has been very recently defined and a role of P27 in the binding to mycobacterial glycolipids that are TLR2 agonists has been proposed [11]. In agreement with the hypothesis that lipoproteins participate in the cell wall integrity, Banaei *et al *[17] found that the lack of LspA expression in *M. tuberculosis *causes higher susceptibility to malachite green and higher decolorization of this compound in the presence of the bacterium. These authors proposed that defects in cell wall permeability are responsible for the hypersensitivity of the *lspA *mutant to malachite green. We found that in the absence of *P27-P55 *expression, *M. tuberculosis *is extremely sensitive to malachite green and that the mutant decolorized malachite green faster than the wild type. The expression of P55 alone, but not of P27, in the mutant strain partially restored the wild type level of resistance to malachite green, supporting the idea that P55, through an efflux system is mainly involved in this phenomenon. Unfortunately, the addition of reserpine to strain cultures blocked the decolorization process, thus not allowing us to assess the effect of this drug in the efflux pump activity encoded in the *P27-P55 *operon.

The higher susceptibility to isoniazid and ethambutol of the MtΔP27 mutant is in agreement with a previous publication in which an increased expression of P55 was detected in the presence of isoniazid in a multidrug-resistant *M. tuberculosis *strain [26]. Moreover, the involvement of P55 in the mechanism of antibiotic resistance has been demonstrated in *Mycobacterium bovis *BCG [6]. Based on these results, which demonstrate that a P55-knockout *M. bovis *BCG strain is more susceptible to rifampicin, ethambutol and other drugs, Ramón-García et al. proposed that P55 plays an essential role of the efflux pump in detoxification processes coupled to oxidative balance within the bacterium. Consistently with that, here we found that both P27 and P55 provide *M. tuberculosis *with resistance to ethambutol; however, the susceptibility of *M. tuberculosis *to rifampicin was unchanged in the MtΔP27 mutant, suggesting that the intrinsic mechanism of antibiotic resistance is determined by the genetic background of the bacterial species.

The high susceptibility of MtΔP27 to ethidium bromide also supports a role of P27 and P55 in efflux transport. Farrow and Rubin have demonstrated that P55 mediates the transport of ethidium bromide outside *M. smegmatis *and that P27 is required for this process [10]. The finding that reserpine alters the intrinsic resistance of *M. smegmatis *[10] and *M. tuberculosis *(this study) to ethidium bromide indicates that the susceptibility observed in the MtΔP27 mutant is due to the lack of the efflux pump function rather than to an increase in cell wall permeability. This assumption is supported by the fact that the introduction of *P55 *into the MtΔP27 mutant showed partial resistance to ethidium bromide.

## Conclusions

Our results showed here demonstrate that P27 and P55 are functionally connected in processes associated with cell wall function by contributing to both the impermeability of the cell wall and the transport of toxic compounds away from the cells. In this regard, it has been proposed that P55 plays a role in the detoxification systems linked to respiratory processes and maintenance of the redox balance within the cell [6]. Ongoing research is aimed to find out the precise role of P27 in these processes.

We propose that the requirement of P27 and P55 for the replication and persistence of the bacterium during the host infection is based on two aspects: the physiological role of P27 and P55 in cell wall function and transport, which are relevant during the *in vivo *growth of *M. tuberculosis*, and the antigenic properties of P27 to exert immune evasion during persistent *M. tuberculosis *infection.

## Competing interests

The authors declare that they have no competing interests.

## Authors' contributions

MVB performed the decolorization assays as well as the SDS and ethidium bromide susceptibility assays. RVR generated the complemented strains. FCB and MAF performed the RT-qPCRs. BI and NM carried out the first-line drug susceptibility testing. AAC and LIK participated in the design of the study. FB conceived the study, participated in its design and coordination, and drafted the manuscript. All authors read and approved the final manuscript.

## Pre-publication history

The pre-publication history for this paper can be accessed here:

http://www.biomedcentral.com/1471-2334/11/195/prepub

## Supplementary Material

Additional file 1 **Transcription of *P55 *in ΔP27 complemented strains**. DNA-free RNA extracted from middle logarithmic-phase cultures of H37Rv, MtΔP27::P55 and MtΔP27::P27-P55 was reverse transcribed using random hexamers as primers. Total cDNA samples were used as template in Q-PCR assays to relatively quantify the number of copies of *P55 *mRNA. Results were presented as relative expression to H37Rv. Click here for file

Additional file 2 **Decolorization of malachite green in MtΔP27 mutant**. Bacterial strains grown to mid-log phase were diluted to an OD 600 nm of 0.40-0.44 in PBS. Bacterial suspensions were centrifuged the absorbance at OD 620 nm was measured in the supernatant at time points indicated. *Significantly different from values of the wild type strain. Click here for file
