# Supplementary material for: Role of P27 -P55 operon from Mycobacterium tuberculosis in the resistance to toxic compounds
Source: BMC Infect Dis. 2011 Jul 16;11:195. doi: 10.1186/1471-2334-11-195 (PMC3146831; doi:10.1186/1471-2334-11-195)
Supplement: Additional file 1 — Transcription of P55 in ΔP27 complemented strains. DNA-free RNA extracted from middle logarithmic-phase cultures of H37Rv, MtΔP27::P55 and MtΔP27::P27-P55 was reverse transcribed using random hexamers as primers. Total cDNA samples were used as template in Q-PCR assays to relatively quantify the number of copies of P55 mRNA. Results were presented as relative expression to H37Rv. [file 1471-2334-11-195-S1.DOCX]

| **Strain** | **Fold change^§^** |
| --- | --- |
| H37Rv  MtΔP27::P55 | 1  55.60 (+/-3.46)* |
| MtΔP27::P27-P55 | 11.32 (+/-1.21)* |

**Transcription of *P55* in ΔP27 complemented strains**

**^§^**Relative expression to H37Rv. Values show means +/- standard deviations of technical duplicates. Significantly different to values of H37Rv (*p* <0.001)**^*^**

DNA-free RNA (1µg) extracted from middle logarithmic-phase cultures [1] was used as template of SuperScript III reverse Transcriptase (Invitrogen) using random hexamers as primers. Identical reactions lacking reverse transcriptase were also performed to confirm the absence of genomic DNA in all samples. Q-PCR assays were performed with Master Mix QuantiTect SYBR Green (Qiagen). Results were presented as ratios calculated with the Relative expression software tool (REST@) application described by Plaffl et al. [M.W. Pfaffl, G.W. Horgan, L. Dempfle, Relative expression software tool (REST) for group-wise comparison and statistical analysis of relative expression results in real-time PCR, Nucleic Acids Res. 30 (2002) e36]. Relative quantification of *P55* was performed by using *sigA* as reference gene and a subsequent test for significance of derived results was performed by using Pair Wise Fixed Reallocation Randomisation. The value of PCR efficiency for all transcripts was 2, as calculated following the formula: E= 10 ^[-1/slope]^.
