# Supplementary material for: Role of P27 -P55 operon from Mycobacterium tuberculosis in the resistance to toxic compounds
Source: BMC Infect Dis. 2011 Jul 16;11:195. doi: 10.1186/1471-2334-11-195 (PMC3146831; doi:10.1186/1471-2334-11-195)
Supplement: Additional file 2 — Decolorization of malachite green in MtΔP27 mutant. Bacterial strains grown to mid-log phase were diluted to an OD 600 nm of 0.40-0.44 in PBS. Bacterial suspensions were centrifuged the absorbance at OD 620 nm was measured in the supernatant at time points indicated. *Significantly different from values of the wild type strain. [file 1471-2334-11-195-S2.PPT]

## Slide 1
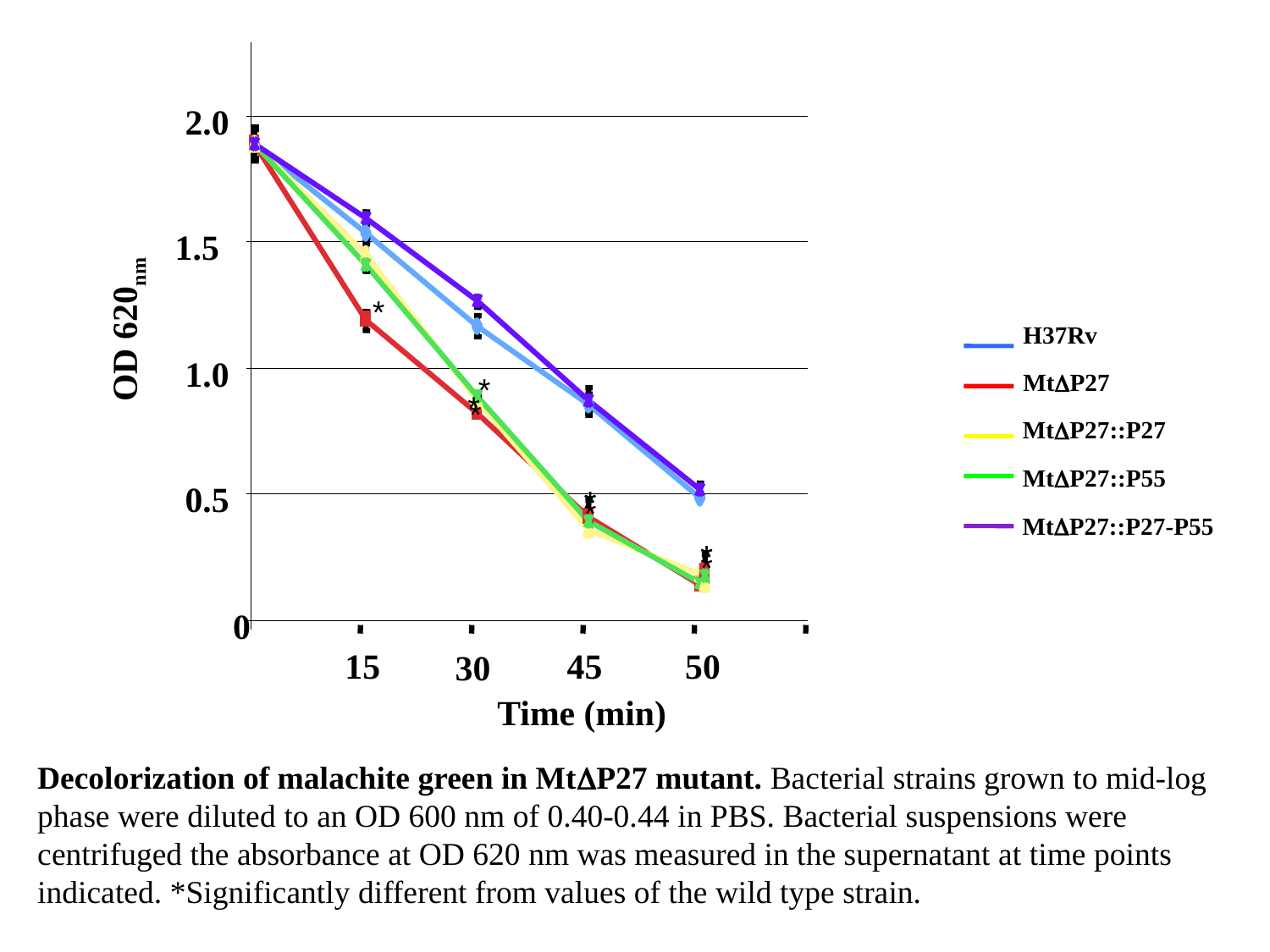

2.0
1.5
OD 620nm
*
H37Rv
1.0
*
MtΔP27
*
*
MtΔP27::P27
MtΔP27::P55
*
*
0.5
*
MtΔP27::P27-P55
*
*
*
0
15
50
45
30
Time (min)
Decolorization of malachite green in MtΔP27 mutant. Bacterial strains grown to mid-log phase were diluted to an OD 600 nm of 0.40-0.44 in PBS. Bacterial suspensions were centrifuged the absorbance at OD 620 nm was measured in the supernatant at time points indicated. *Significantly different from values of the wild type strain.
